# Mathematical modelling of the antibiotic-induced morphological transition of *Pseudomonas aeruginosa*

**DOI:** 10.1371/journal.pcbi.1006012

**Published:** 2018-02-26

**Authors:** Chloe Spalding, Emma Keen, David J. Smith, Anne-Marie Krachler, Sara Jabbari

**Affiliations:** 1 School of Mathematics, University of Birmingham, Edgbaston Campus, Birmingham, United Kingdom; 2 Institute of Microbiology and Infection, School of Biosciences, University of Birmingham, Edgbaston Campus, Birmingham, United Kingdom; 3 Institute for Metabolism and Systems Research, University of Birmingham, Edgbaston Campus, Birmingham, United Kingdom; 4 Department of Microbiology and Molecular Genetics, University of Texas McGovern Medical School at Houston, Houston, Texas, United States of America; Rice University, UNITED STATES

## Abstract

Here we formulate a mechanistic mathematical model to describe the growth dynamics of *P. aeruginosa* in the presence of the *β*-lactam antibiotic meropenem. The model is mechanistic in the sense that carrying capacity is taken into account through the dynamics of nutrient availability rather than via logistic growth. In accordance with our experimental results we incorporate a sub-population of cells, differing in morphology from the normal bacillary shape of *P. aeruginosa* bacteria, which we assume have immunity from direct antibiotic action. By fitting this model to experimental data we obtain parameter values that give insight into the growth of a bacterial population that includes different cell morphologies. The analysis of two parameters sets, that produce different long term behaviour, allows us to manipulate the system theoretically in order to explore the advantages of a shape transition that may potentially be a mechanism that allows *P. aeruginosa* to withstand antibiotic effects. Our results suggest that inhibition of this shape transition may be detrimental to bacterial growth and thus suggest that the transition may be a defensive mechanism implemented by bacterial machinery. In addition to this we provide strong theoretical evidence for the potential therapeutic strategy of using antimicrobial peptides (AMPs) in combination with meropenem. This proposed combination therapy exploits the shape transition as AMPs induce cell lysis by forming pores in the cytoplasmic membrane, which becomes exposed in the spherical cells.

## Author summary

Antimicrobial resistance is an urgent global health threat and it is critical that we formulate alternative treatment strategies to combat bacterial infections. To do this we must understand how bacteria respond to currently used antibiotics. *Pseudomonas aeruginosa* is the leading cause of death among cystic fibrosis patients, a top cause of hospital-acquired infections in the UK and is currently listed as a critical priority in a list of antibiotic-resistant bacteria produced by the World Health Organisation. *P. aeruginosa* can change shape in the presence of certain antibiotics that work by targeting cell wall synthesis. The bacteria make the reversible transition from the native rod shape to a fragile spherical shape by shedding the cell wall and in doing so they evade the effects of the antibiotic. We formulate a system of equations that describes the growth of the bacteria including the shape transition we witness when we add antibiotic. Fitting this model to experimental data, we obtain parameter values that we then vary to make predictions on how inhibiting the shape transition or increasing the death rate of spherical cells would affect the overall bacterial growth. These predictions can support suitable combination therapies and hint towards alternative treatment strategies.

## Introduction

Antimicrobial resistance (AMR) is now acknowledged as an urgent global health threat and the severity of the situation was highlighted by the World Health Organization 2014 report that discusses the increasing incidence of resistance-induced health problems in every region of the world [1]. A “post-antibiotic” era is described, where even a simple infection can become fatal as current drug strategies fail to ameliorate previously manageable infections.

It is imperative that we try to gain a deeper understanding of currently used drug treatments and specifically the mechanism of action of a drug and the consequential response of a bacterial population. Elucidating the mechanistic interactions between bacteria and antibiotic increases our understanding of how pathogens react in response to antimicrobials and the concurrent impact on the selective pressure that can influence the emergence of resistance. A popular strategy used to investigate mechanisms of action is the examination of the morphology of treated bacteria. This is a relatively simple experimental procedure that can be used as an initial preliminary step in an investigation or to provide further evidence to support a suspected mechanistic interaction.

Investigation into bacterial response has shown that many bacteria undergo changes in their morphology as a result of antibacterial action. Morphological changes such as filamentation (cell elongation), localised swelling and bulge formation can often be attributed to specific antibiotic mechanisms of action [2]. For example, antibiotic agents that alter lateral cell wall synthesis by disrupting the peptidoglycan-synthesizing enzymes can often cause cells to decrease in length, producing ovoid cells [3, 4]. Observations like this can be the result of multiple mechanistic interactions between the antibiotic and the bacteria and it can also be difficult to differentiate between changes in morphology. Many of these structural changes may occur to varying extents depending on factors such as the antibiotic concentration, incubation conditions and how long the bacteria is exposed to the agent [2, 5]. Although this can result in structural heterogeneity within bacterial populations, any observations of changes in cellular morphology can still act as an indication of the occurrence of a certain mechanism of action.

One bacterial species that shows significant changes to its morphology in response to antimicrobials is *Pseudomonas aeruginosa*, a Gram-negative pathogenic bacteria that also shows notable levels of resistance to many antibiotics. This extremely versatile, opportunistic pathogen is able to acquire nutrients from a wide range of organic matter, meaning that it can easily infect damaged tissues in animals and humans. *P. aeruginosa* is an example of a nosocomial pathogen, a characteristic that arises from its ability to survive in moist environments and on hospital instruments such as catheters. Infections are often found in airways, urinary tracts and in burns and wounds and can often be asymptomatic until a biofilm forms. This can overwhelm the immune system and cause bacteraemia, pneumonia and sepsis, and can ultimately lead to death; this makes *P. aeruginosa* especially threatening to those who are immunocompromised, including in particular patients with cystic fibrosis [6].

There are several antibiotics that still have activity against *P. aeruginosa* including some carbapenems, a class of *β*-lactams, the most widely used group of antibiotics. Identified by possessing a *β*-lactam ring, this class of antibiotics inhibits cell wall synthesis by binding to penicillin binding proteins (PBPs). Inhibition of cell wall synthesis results in structural changes to the bacteria and ultimately this leads to lysis. Some strains of *P. aeruginosa* display resistance to *β*-lactam antibiotics, for example through the production of *β*-lactamases and the upregulation of efflux pumps. Additionally, there is evidence that some genetically susceptible strains are able to tolerate the presence of many *β*-lactams, including meropenem, for periods of time.

Meropenem, a carbapenem that has been shown to have greater antibacterial action against *P. aeruginosa* compared to other carbapenems, induces several morphological changes in this bacteria including filamentation and spheroplast formation [7]. Its enhanced antibacterial activity and the varying resultant responses in morphology are often attributed to its affinity for both PBP2 and PBP3 enzymes, which lead to inhibition of peptidoglycan synthesis at different regions of the bacterial wall.

The work of Monahan *et al*. [8] investigates the tolerance of *P. aeruginosa* to *β*-lactam antibiotics, including meropenem, and suggests that the ability of the bacteria to withstand high concentrations of *β*-lactams can be attributed to a rapid transition in cell morphology. This population-wide change, activated by antibiotic exposure, is described as a survival strategy that results in the bacteria evading antibiotic effects. The experimental results show that antibiotic exposure induces a transition in morphology that produces a subpopulation of spherical cells that possess a defective cell wall. These spherical cells are thought to be able to survive antibiotic effects since *β*-lactam drugs target the ability of the bacteria to synthesise its cell wall. It was first thought that the cell wall deficient spherical cells would lyse and not be viable due to the delicate structure of the cell, however, it was found that not only did the whole population of cells transition from rod to spherical-shaped within 24 hours, but also 84% of these cells were viable and able to transition back to rod-shaped cells in the absence of the meropenem [8].

This population of spherical shaped cells can therefore evade the effects of the antibiotic and lay dormant until the antibiotic is no longer a threat, at which point they possess the ability to transition back into rod-shaped cells and resume proliferation. Whether this large scale transition is an observation of the mechanistic interactions between the drug and the bacteria or a mechanism used as a defensive strategy controlled by the bacteria, the viability of these cells and the possibility of a reverse transition could explain some of the persistent characteristics of *P. aeruginosa*. *Persistence* is described as a result of phenotypic heterogeneity and this phenomenon occurs due to the formation of a subpopulation of persister cells with potentially different growth and survival rates. These cells, often developing due to environmental stresses, lay dormant until more favourable conditions occur; at which point, the possibility of reversion results in persistent infections that do not necessarily rely on resistance genes. The varying characteristics of the subpopulations we see when meropenem is added to *P. aeruginosa* form an analogous environment.

The task of providing new treatment strategies and exploring the interactions between drugs and bacteria has traditionally fallen to the pharmaceutical industry but with the waning effectiveness of classic antibiotic strategies, it is imperative that we can harness ideas and techniques from other disciplines in order to explore how we can control antibiotic resistance. Mathematical modelling and in particular the study of population dynamics, is increasingly being used to study the changes in growth of bacterial populations, the effects of antibiotics and the emergence of resistance.

Here we develop a system of differential equations in order to describe the bacterial growth of *P. aeruginosa* in the presence of the antibiotic meropenem with the inclusion of a morphological transition that is activated by the presence of the antibiotic. Whether this transition is a result of direct antibiotic action or attributable to an intrinsic survival mechanism, we assume that these cell wall deficient spherical cells evade antibacterial effects, as evidenced by the data in [8] and our own data. The model is formulated to to be entirely mechanistic with growth depending explicitly on nutrient availability. Using *in vitro* data of growth curves of *P. aeruginosa* in the presence of varying concentrations of the antibiotic meropenem, we obtain parameter estimates for the model that enable it to reproduce experimentally observed behaviour. Analysis of the results to changes in these parameters allows us to explore the effects of the shape transition on bacterial susceptibility to meropenem. We note that our investigation will focus solely on a meropenem-susceptible strain of *P. aeruginosa*; our concern is not with the mechanisms of resistance that are often attributed to *P. aeruginosa*, such as the production of *β*-lactamases or the upregulation of efflux pumps. Rather, we wish to investigate a shape transition that permits meropenem-susceptible strains of *P. aeruginosa* to tolerate the presence of antibiotics.

## Materials and methods

### Staining and fluorescence microscopy

Single colonies of the *P. aeruginosa* strain PA1008, a meropenem susceptible isolate derived from a hospital burn unit [9], were inoculated into LB broth and grown for around 16 hours at 37°C, shaking at 200 rpm.

#### Microscopy images for displaying transition

Cultures were normalised to an optical density at 600 nm (OD_600_) of 0.2 in 20 ml LB broth containing the desired concentration of meropenem. Cultures were incubated at 37°C, 200 rpm in between time points.

At each time point, 1 ml of bacteria was removed from each flask and the OD_600_ measured. A sample from each flask was then normalised to an OD_600_ of 1 by centrifuging the sample and re-suspending in the appropriate volume of PBS. 500*μ*l of each sample was stained with 1.5*μ*l of LIVE/DEAD BacLight stain mixture and incubated for 15 minutes at room temperature, in the dark. Samples were centrifuged and re-suspended in 4% PFA for 10 minutes at room temperature to fix the samples. Samples were washed three times with PBS to remove the fixative, and re-suspended in 500*μ*l of PBS following the final wash.

5*μ*l of stained culture was loaded onto a microscope slide and allowed to air dry. Once dry, one drop of antifade gold mounting solution was loaded onto the sample and covered with a cover slip. These were left overnight to set in the dark. Microscope slides were examined using a Zeiss Axio Observer microscope. Images were taken using the 63× oil immersion objective and exposure to transmitted light, red and green fluorescence. For fluorescence an exposure of 500 ms was used.

#### Microscopy images for parameter testing

Cultures were normalised to an optical density at 600 nm (OD_600_) of 0.4 in LB broth after 16 hours of growth. Meropenem dilutions were made at twice the desired final concentration in LB. 50*μ*l of the normalised PA1008 culture was added to a 96-well plate followed by 50*μ*l of the meropenem dilution. For each condition, 6 wells were prepared so that the contents could be pooled together to increase the yield of cells. Between time points the 96-well plate was incubated in a plate reader, shaking at 200 rpm, 37°C. OD_600_ was measured from the samples every 10 minutes to allow the OD of the samples to be monitored over the course of the experiment (thus ensuring bacterial load is comparable to that of our growth curves—see below).

At each time point, the contents of the appropriate wells were removed and pooled together for each concentration in separate microcentrifuge tubes. 600*μ*l of 4% PFA was added directly and incubated for 30 minutes at room temperature to fix the samples. Samples were washed three times with PBS to remove the fixative, and re-suspended in 50*μ*l of PBS following the final wash. Each sample was stained with 3*μ*l of LIVE/DEAD BacLight stain mixture and incubated for 15 minutes at room temperature, in the dark. Samples were washed three times with PBS to remove excess stain, and re-suspended in 50*μ*l of PBS following the final wash.

5*μ*l of stained culture was loaded onto a microscope slide and allowed to air dry. Once dry, one drop of antifade gold mounting solution was loaded onto the sample and covered with a cover slip. These were left overnight to set in the dark. Microscope slides were examined using a Zeiss Axio Observer microscope. Images were taken using the 63× oil immersion objective and exposure to transmitted light, red and green fluorescence. For fluorescence an exposure of 100 ms was used. At least 5 images were obtained for each condition.

### Growth curves for model parametrisation

Single colonies of the *P. aeruginosa* strain PA1008 were inoculated into 5 ml of LB broth and grown overnight at 37°C, shaking at 200 rpm. The following day, the cultures were diluted to an OD_600_ of 0.4, which would be diluted to 0.2 following the addition of the antibiotic. Dilutions of meropenem were made using LB broth to give final concentrations. 50*μ*l of bacteria culture was added to the appropriate wells of a sterile, 96 well, flat bottom microtitre plate, followed by 50*μ*l of the appropriate meropenem dilution. Media controls were then added to the plate for each meropenem concentration, consisting of 50*μ*l of each meropenem dilution plus 50*μ*l of LB broth. The OD_600_ of each well was taken using a FLUOstar Omega plate reader every 30 minutes for 24 hours. Between OD readings the plate was shaken for 27 minutes. The temperature was set to 37°C for the duration of the assay.

In order to successfully parametrise the model of Eqs 2–6 we have used two sets of data, which we will refer to as data set 1 and data set 2. The first data set is used as parametrisation data; this consists of three biological replicates, each with three technical repeats. The meropenem concentrations used for this data set were 0*μ*g ml^−1^, 2*μ*g ml^−1^, 4*μ*g ml^−1^, 10*μ*g ml^−1^, 20*μ*g ml^−1^, 40*μ*g ml^−1^ and 200*μ*g ml^−1^. For each concentration we display the mean of the nine resulting growth curves. The error bars are formulated by calculating the standard deviation of the means of the three biological replicates.

Data set 2 is also referred to as the test data and includes one biological replicate with three technical repeats; the meropenem concentrations used for data set 2 were 0*μ*g ml^−1^, 0.5*μ*g ml^−1^, 1*μ*g ml^−1^, 5*μ*g ml^−1^, 50*μ*g ml^−1^ and 100*μ*g ml^−1^. For each concentration, we calculate the mean value of the three resulting growth curves at each time point and the standard deviation error bars for this data set represent the standard deviation over the three technical repeats.

### Parameter fitting methods

The parameter estimates were obtained using the MATLAB function *fminsearch*, which uses the Nelder-Mead algorithm as described in [10]. The *fminsearch* function fits parameters that minimise a specified objective function given some initial estimates. We employ the following objective function,

$$\begin{array}{c} R=\frac{1}{n}(\frac{\left|\right|(H+\frac{S}{2})-y{\left|\right|}_{2}}{{\left||y|\right|}_{2}}). \end{array}$$ (1)

The vectors ***H*** and ***S*** are the solutions for rod-shaped and spherical cells respectively at the time points used in the experiments. The vector ***y*** consists of the OD values obtained in the experiment and *n* is the number of data values. This equation evaluates the mean relative error of the difference between the data and the solution to the system at each time point. We use the relative error in our objective function as unlike the absolute error the relative error should not be skewed by large or anomalous data points. We note here that the contributions of rod and spherical shaped cells to the OD differ in the objective function. OD is a measurement of the absorbency of a material; it measures how much light can pass through a sample and therefore reflects the total number of cells present in the inoculum. Rod and spherical cells will contribute differently to the OD measurement as they differ in size and absorbency. Therefore, rather than attempt to scale OD to obtain a total cell number, we work in OD and based on our biological insight we have assumed that spherical cells contribute half the absorbance of rod cells to the OD measurements.

When using the MATLAB function *fminsearch* to parametrise we also apply a constraint that restricts parameter values to non-negative values. To do this we apply a penalty to the objective function that increases its value significantly if a parameter is less than zero; we simply multiply the objective function value by 1000 (i.e. we make the penalised objective function much larger than we anticipate the relative error using the fitted parameters to be).

### Model formulation

Deterministic ODE models are commonly used to model bacterial growth and generally use mass action kinetics to assign rates of biological processes to the species involved. Bacterial growth can be described using a variety of growth curves outlined in [11], with arguably the most commonly used being the logistic growth curve, proposed by Verhulst in 1845. Logistic growth describes growth at a rate that decreases, and ultimately ceases, as the population size reaches a saturating carrying capacity [12–15]. This incorporates competition for resources by making the rate of growth dependent on the relation between the current population and the saturating population constant. This means that it does not explicitly take into account the nutrient available to the bacteria for growth; this can be incorporated using a density dependent rate, such as Monod kinetics [16]. Using Monod kinetics to model bacterial growth provides a more mechanistic approach that offers more meaningful constants as opposed to the phenomenological nature of the logistic equation.

Antimicrobial action can be incorporated using well established concepts from pharmacokinetics and pharmacodynamics. Antibiotic degradation is most commonly modelled via first order kinetics [17–19] and antibiotic effects are often modelled using a density dependent term that will saturate as a consequence of limited binding receptors. Other notable examples of mathematical modelling in bacterial growth and antibiotic action can be seen in [12, 20–29]. Previous models that include subpopulations of persister cells have been used to investigate the growth dynamics of bacteria and the effects of dosing regimens on the persister population, for example [30–32]. In [30], a simple mathematical model is formulated using quantitive measurements to describe persistence of single cells of *Escherichia coli*. The results highlight the need to consider persistent subpopulations and suggest their potential as treatment targets. A general model for persister formation is developed in [31] and is used to investigate the effectiveness of a periodic dosing regime and explore the impact the regime has on the persister population. The investigation in [32] follows on from [31], yet this model is extended by assuming that persister formation occurs due to the production of toxins within the bacteria. The model we will develop will incorporate the specific shape transition witnessed in *P. aeruginosa* population dynamics to explore the persistent nature of the cell wall deficient spherical cells.

**Fig 1:**
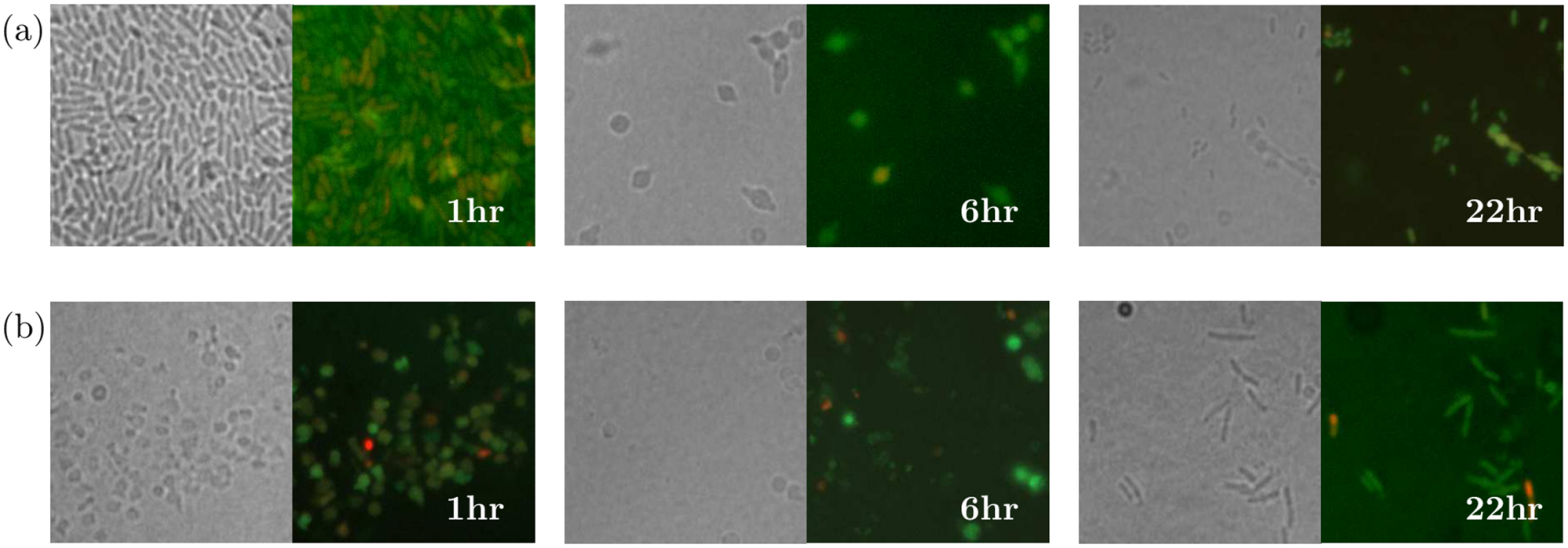
*P. aeruginosa* cells transitioning between a rod and spherical shape. *P. aeruginosa* cells rapidly convert to a spherical shape after adding (a) 0.5*μ*g ml^−1^ and (b) 2*μ*g ml^−1^ of meropenem before reverting back to a bacillary form. Microscopy images were taken after 1, 6 and 22 hours. Left panels: transmitted light, right panels: fluorescence microscopy. In fluorescence images, green and red colouring indicates viable and lysed cells respectively.

The assumptions made for the model are based upon the results in [8], which we have reproduced experimentally, with our results displayed in Fig 1. The images indicate changes to cell morphology shortly after exposure to the antibiotic; spherical shaped cells can be seen after only one hour. After 22 hours we see rod-shaped cells that are in the process of dividing (Fig 1(b)) and this indicates that the cells can revert back from the spherical form and continue proliferation. Additionally, the fluorescence microscopy results support the results of [8]; most of the cells stained green, indicating that these spherical forms were viable.

**Fig 2:**
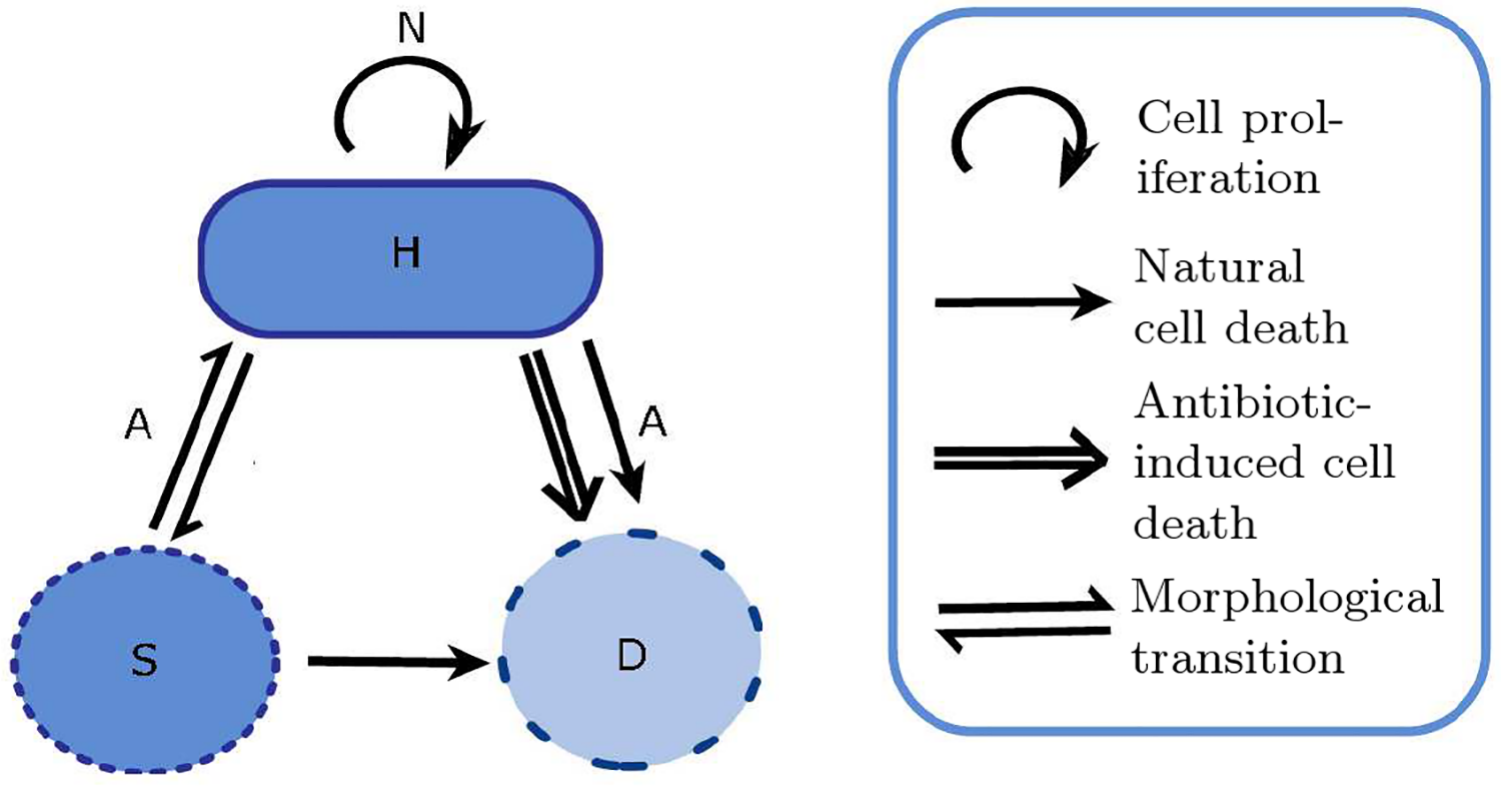
Transitions of *P. aeruginosa* in response to exposure to meropenem. The species shown are defined as follows: *H* are rod-shaped cells, *S* are spherical cells and *D* represent dead cells. When the antibiotic, *A*, is introduced, rod-shaped cells can make the reversible transition to a spherical shape. The antibiotic is assumed to be effective at killing rod-shaped cells but not spherical cells due to a depleted cell wall. Rod-shaped cells lyse naturally as do spherical cells. However, natural rod-shaped cell death is expected to happen at a slower rate than death due to antibiotic action; the double-lined arrow from *H* to *D* represents antibiotic-induced death. Rod-shaped cells can proliferate if there is enough nutrient, *N*.

In accordance with these findings, our compartmental model follows the interactions described in Fig 2. We introduce variables (defined in Table 1) for rod-shaped bacteria (*H*) and spherical-shaped bacteria (*S*). Here, we assume that the rod-shaped cell population consists of all those cells that are susceptible to the antibiotic, this may include cells that have shown signs of localised swelling or filamentation. In this model we will refer to this population as rod-shaped whereas the spherical cell population is made up of cells that have transitioned into this form due to antibiotic exposure. Experimental data has shown that the spherical cells are susceptible to lysis due to internal turgor pressure and that this causes the cells to expand before they lyse. For this model we will contain all spherical cells, regardless of their size, in the same population.

**Table 1:** Definitions and units for the variables in the model, Eqs (2)–(6).

Variable	Definition	Units
*H*	Antibiotic-susceptible rod-shaped cells	OD
*S*	Spherical shaped cells	OD
*N*	Nutrient proportion of initial concentration	dimensionless
*A*	Antibiotic concentration	*μ*g ml^−1^

In addition to these variables we define the antibiotic concentration to be *A* and the proportion of nutrient remaining in the system, *N*. Including a nutrient dependent growth term, instead of the commonly used logistic growth term, will allow us to formulate a mechanistic model. Fig 2 also includes a population of dead cells, however we do not include this variable in the model or results. Any lysed cells will quickly disappear from the culture, we can therefore assume that the contribution made to the optical density by the lysed cells can be considered negligible. This assumption is supported by Fig 1; with only a minority of cells in the image fluorescing red (indicating a dead cell) we can assume that the majority of dead cells have completely lysed and disappeared.

We assign parameters for the rates of growth, death and transition as defined in Table 2. Nutrient dependent growth will occur at the rate *r* and proliferation will concurrently incur a decrease in nutrient that occurs at the rate $\tilde{r}$. For this model we assume that only rod-shaped cells can proliferate. This assumption is based on existing literature, experimentally characterising the proliferation of usually rod-shaped bacteria that have undergone conversion to spherical cells as a result of shedding the cell wall [33]. The experiments demonstrated that although proliferation of spherical cells was possible, it was only possible under specific osmoprotective conditions and even then proceeds in only a very small subset of spherical cells, with a doubling time 10-fold higher than that of the rod-shaped cells. Conventional proliferation becomes impossible due to the compromised integrity of the spherical cell wall; bacterial proliferation is heavily dependent on correct positioning of the cell wall and without this it is unlikely that a cell will successfully divide. Although *P. aeruginosa* was not included in these experiments, it was suggested that similar proliferation occurs across all bacteria; due to the high doubling time we will assume that spherical growth is negligible.

**Table 2:** Definitions and units for the parameters in the model, Eqs (2)–(6).

Parameter	Description	Units
*α*	Antibiotic decay rate.	min^−1^
*r*	Growth rate of rod-shaped bacteria.	min^−1^
$\tilde{r}$	Decay rate of nutrient due to consumption by rod-shaped bacteria.	OD^−1^ min^−1^
*γ*	Transition rate from rod to spherical shape.	min^−1^
*δ*	Transition rate from spherical to rod shape.	min^−1^
*ρ*	Death rate of rod-shaped bacteria due to antibiotic.	min^−1^
$\tilde{\rho }$	Decay rate of antibiotic due to irreversible binding to bacteria.	*μ*g ml^−1^ OD^−1^ min^−1^
*ϕ*	Death rate of rod-shaped bacteria.	*min*^−1^
*ψ*	Death rate of spherical bacteria.	*min*^−1^
*T*_50_	Antibiotic concentration required for half maximal killing effect	*μ*g ml^−1^
*A*_50_	Antibiotic concentration required for half maximal transition effect	*μ*g ml^−1^

Meropenem is bactericidal hence its action is modelled via a death term distinct from natural death [34]. This additional term differs from some models that absorb the antibiotic effects into the model by means of a reduction in bacterial growth, yet this approach would be more suited to bacteriostatic antibiotics [17, 25]. The antimicrobial effects on the rod-shaped population is modelled using a density dependent term to incorporate a saturating effect. Attempts at using a linear term for this resulted in unsuccessful parameter fits (results omitted) and a saturating term was found to be more suitable in describing the effects of the antibiotic; this is in keeping with previous models of antibiotic treated bacteria [14, 17, 35]. We assume that the meropenem only inhibits rod-shaped cell survival as experimental results indicate that transitioning to a spherical form mediates tolerance to lethal *β*-lactam antibiotic concentrations for multiple bacterial species [8, 33]. We assign *ρ* to be the maximum death rate of rod-shaped cells due to antibiotic and *A*_50_ to be the antibiotic concentration needed for half maximal killing. We assume that the meropenem molecules will enter the cell and bind to the PBPs in order to inhibit cell wall synthesis. In this model we assume that this binding is irreversible due to mode of action of *β*-lactams. The antibiotic molecules covalently modify PBPs upon binding [36] and therefore when a bacterium lyses, any bound antibiotic is unlikely to be released back into the extracellular domain. The irreversibility of the antibiotic results in antibiotic decay and we will take this to happen at a rate, $\tilde{\rho }$. Investigation into this parameter has shown that relaxing this parameter and setting $\tilde{\rho }=0$ *μ*g ml^−1^ OD^−1^ min^−1^ does not affect the results significantly (results omitted) yet we include this assumption as it has an impact on long-term model predictions of bacterial growth. In addition to this, we take antibiotic decay to follow first order kinetics at a rate *α*. In concordance with [8], we assume that spherical cells evade the effects of the antibiotic.

The primary objective of this model is to investigate whether we can describe the population dynamics of *P. aeruginosa* in the presence of meropenem by including the morphological transition witnessed in experimental data. We model this transition from rod-shaped cells to spherical-shaped cells with a saturating term, similar to that used for the bactericidal effects of the antibiotic on the rod-shaped cell population. Following exposure to the meropenem the rod-shaped cells transition to a spherical form at a maximum rate *γ* and *T*_50_ defines the concentration for which we get the half maximal transition rate. Following [8, 33] we include the process of reversion within our model: antibiotic is removed from samples in order to experimentally show that spherical cells can transition back to the bacillary form, implying that this occurs independently from the presence of the antibiotic. Previous models of persister populations have assumed that reversion does not occur when a constant dose of antibiotic is administered [31, 32]. However, in our model, antibiotic may degrade out of the system and even when present the spherical cells may not be directly exposed to antibiotic at all times. Reversion will be independent from the antibiotic concentration and we define *δ* to be the rate at which this occurs. Supported by the microscopy results in Fig 1, this reverse transition also lends weight to the assumption that antibiotic decays as it suggests that the pressure on the bacteria from the antibiotic has decreased.

Finally, following our own fluorescence microscopy data, we assume that both types of cell will lyse naturally. In our microscopy results we witnessed lysed (red) rod-shaped cells even in the absence of antibiotic, indicating that some level of natural death must occur. Subsequently, we assume that spherical cells also lyse naturally due to the increased fragility of the cell structure. Lysed cells degraded sufficiently quickly that we may assume they do not contribute to the OD settings in our experimental set-up. The spherical cells lyse at a rate *ψ*, whilst rod-shaped bacteria lyse at a rate *ϕ*. Due to the availability of nutrient and general fitness of the rod-shaped cells we would expect *ϕ* to be small and due to the compromised integrity of the cell wall in spherical cells we expect *ϕ* < *ψ*.

Following mass action kinetics we produce the following model:

$$\begin{array}{ccc} \frac{dH}{dt} & = & rNH-(\frac{\gamma A}{T_{50}+A})H+\delta S-(\frac{\rho A}{A_{50}+A})H-\phi H, \end{array}$$ (2)

$$\begin{array}{ccc} \frac{dS}{dt} & = & (\frac{\gamma A}{T_{50}+A})H-\delta S-\psi S, \end{array}$$ (3)

$$\begin{array}{ccc} \frac{dA}{dt} & = & -\alpha A-(\frac{\tilde{\rho }A}{A_{50}+A})H, \end{array}$$ (4)

$$\begin{array}{ccc} \frac{dN}{dt} & = & -\tilde{r}NH, \end{array}$$ (5)

with initial conditions

$$\begin{array}{c} H\left(0\right)=H_{0},\;S\left(0\right)=0,\;A\left(0\right)=A_{0},\;N\left(0\right)=1. \end{array}$$ (6)

As *N* is defined to be the proportion of initial nutrient remaining instead of a concentration we can specify *N*(0) = 1. All variables and parameters are defined in Tables 1 and 2 respectively.

## Results

### Model parametrisation

#### Parametrisation without meropenem

We first fit the non antibiotic-associated parameters that we expect to remain fixed in the presence of antibiotic. By removing the antibiotic from the system we are left with the following pair of equations

$$\begin{array}{ccc} \frac{dH}{dt} & = & H(rN-\phi ), \end{array}$$ (7)

$$\begin{array}{ccc} \frac{dN}{dt} & = & -\tilde{r}NH, \end{array}$$ (8)

with initial conditions

$$\begin{array}{c} H\left(0\right)=H_{0},\;N\left(0\right)=1. \end{array}$$ (9)

**Fig 3:**
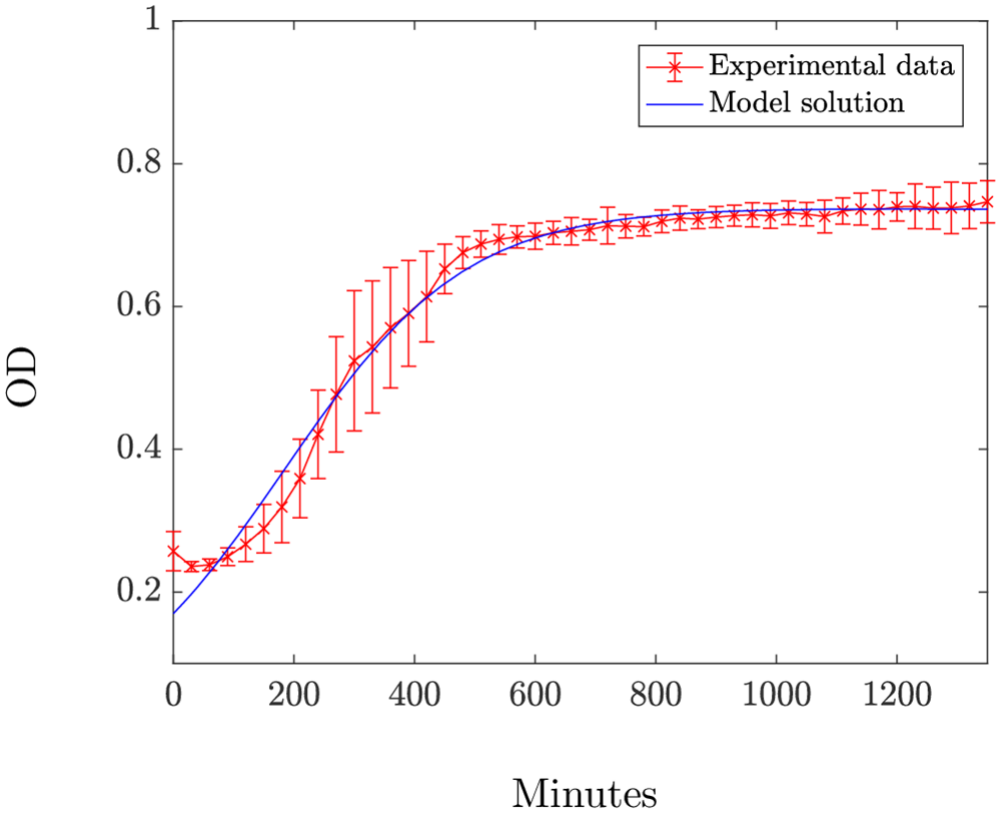
The solution to the system (7)–(9) using the parameters in (10), plotted with the experimental data for the growth curve of *P. aeruginosa* (Data set 1). Data points are plotted with errorbars representing the standard deviation of the means of the three biological replicates.

The initial guess for *r* is 1 × 10^−3^ min^−1^ and for $\tilde{r}$ we choose 1 × 10^−1^ OD^^1^^ min^−1^. The natural death rate, *ϕ*, is assumed to happen at a slower rate than growth; a bacterial population would not thrive if growth and death occurred at the same magnitude and we therefore choose an initial parameter guess for *ϕ* of 1 × 10^−5^ min^−1^. Including the initial condition, *H*_0_, in our estimation allows for the incorporation of error, whether it be systematic or random, within the initial data values. The initial guess for *H*_0_ is chosen to be the initial data value. We attain the results shown in Fig 3 parametrised by the resulting final estimates,

$$\begin{array}{c} \left[r,\;\tilde{r},\;\phi ,\;H_{0}\right]=\left[5.1\times 10^{-3},\;8.9\times 10^{-3},\;7.8\times 10^{-6},\;0.17\right]. \end{array}$$ (10)

All parameters are given to two significant figures and the mean relative error is 7 × 10^−4^. Fig 3 indicates that these parameters produce a good fit and that this model with nutrient dependent growth can describe the growth dynamics of the bacteria in the experiment. We note that the model is unable to reproduce the slight lag phase (see papers by, for example, Zwietering *et al*. and Biranyi *et al*. for developments of the logistic model that can achieve this [11, 37]) and instead underestimates the initial condition; we do not anticipate that this will alter any of the findings of this study and therefore choose not to include further parameters to obtain the lag phase. We also notice that the death rate is much lower than the growth rate; this supports the hypothesis that the natural death rate of the rod-shaped bacteria will be low.

#### Parametrisation with meropenem

Fixing the parameters given by Eq (10), we use the killing curves to estimate the remaining parameters that are attributed to the presence of antibiotic. This data includes the addition of meropenem at multiple concentrations and therefore the full system, Eqs (2)–(6), is used. Early parametrisation attempts provided initial parameter guesses for the optimiser and these values are shown in Table 3 along with the estimated parameter values obtained.

**Table 3:** Estimated parameter values (EPVs) for the model, Eqs (2)–(6).

Parameter	IPG**	EPV	
		Parameter set to incur population elimination Θ_1_	Parameter set to incur population recovery Θ_2_
*r*	1 × 10^−3^	5.1 × 10^−3^	5.1 × 10^−3^
$\tilde{r}$	1 × 10^−3^	8.9 × 10^−3^	8.9 × 10^−3^
*ϕ*	1 × 10^−5^	7.8 × 10^−6^	7.8 × 10^−6^
*α*	1 × 10^−3^	1.4 × 10^−8^	5.5 × 10^−6^
*γ*	3 × 10^−3^	5.4 × 10^−3^	5.4 × 10^−3^
*δ*	1 × 10^−3^	1.8 × 10^−3^	1.8 × 10^−3^
*ψ*	9 × 10^−5^	1.2 × 10^−5^	1.2 × 10^−5^
*ρ*	1 × 10^−3^	1.7 × 10^−3^	1.7 × 10^−3^
$\tilde{\rho }$	1 × 10^−3^	2.9 × 10^−3^	8.2 × 10^−3^
*T*_50_	0.5	0.47	0.47
*A*_50_	0.5	0.13	0.13
R*	-	3.47 × 10^−3^	3.56 × 10^−3^
*OD* when *A*_0_ = 2 at *t* = 5000		0.069	0.37

We define two parameter sets, one that results in population elimination for all concentrations of meropenem (Θ_1_) and another that displays population recovery of the bacteria when 2*μ*g ml^−1^ is added, Θ_2_ (only *α* and $\tilde{\rho }$ differ between the two sets).

Units for each parameter are shown in Table 2.

* *R* represents the sum of the relative errors for each of the separate data sets.

** Early parametrisation attempts were used to gauge reasonable initial parameter guesses (IPGs).

The optimiser estimated the values of all but two parameters, *α* and $\tilde{\rho }$, which were determined to be unidentifiable. These two parameters represent the natural decay rate and the rate of decay due to internalisation of the antibiotic; both are rates of decay of a species for which we do not have data explicitly and thus it is unsurprising that they cannot be identified. Latin hypercube sampling was used to investigate the possible values of the two unidentifiable parameters and the results of this concluded that multiple pairings of values can be used to successfully describe the population dynamics of all concentrations. Further investigation into these feasible parameter pairings revealed two qualitatively different predictions for population growth when the model is simulated over a longer time period than used in experimental procedure. We choose two parameter sets Θ_1_ and Θ_2_ to represent each of these outcomes, see Table 3. The estimated initial condition *H*_0_ obtained for each parameter set can be found in Table 4.

**Table 4:** Estimated initial condition *H*_0_ for each experimental data set.

Antibiotic concentration*	IPG**	EPV***	
		Parameter set Θ_1_	Parameter set Θ_2_
2	0.25	0.23	0.22
4	0.26	0.23	0.23
10	0.26	0.23	0.23
20	0.26	0.23	0.23
40	0.26	0.24	0.24
200	0.25	0.24	0.24

* Initial antibiotic concentration is fixed.

** The initial experimental data values are chosen as the IPGs.

*** EPVs obtained by treating the initial condition as a parameter and simultaneously fitting all data sets, with parameters fixed, using the built in MATLAB function *fminsearch*.

**Fig 4:**
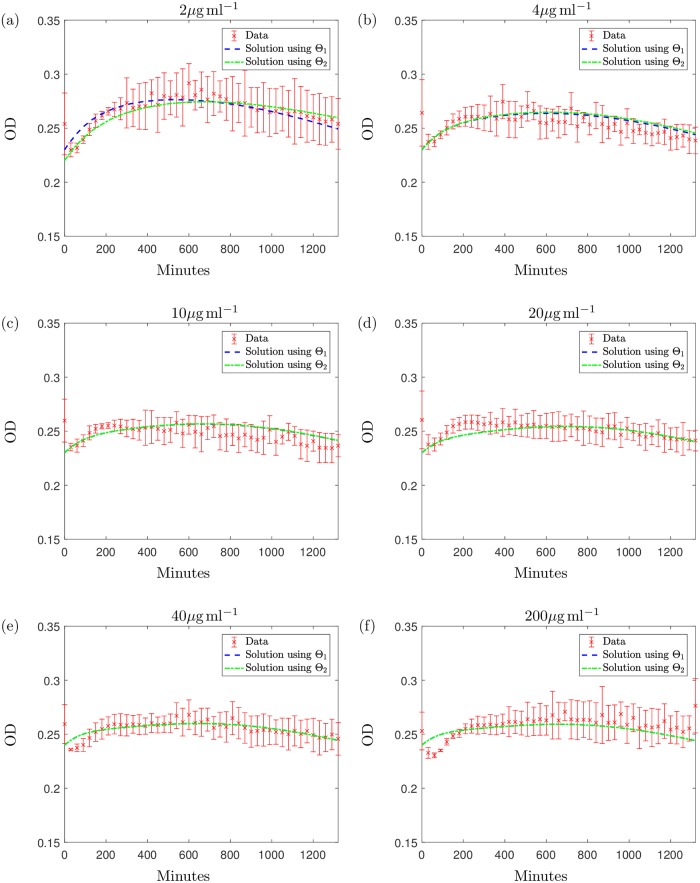
The solution to the system (2)–(6) with Θ_1_ (blue dashed line) and Θ_2_ (green dotted line). Initial conditions are taken from Table 4, for (a) 2*μ*g ml^−1^, (b) 4*μ*g ml^−1^, (c) 10*μ*g ml^−1^, (d) 20*μ*g ml^−1^, (e) 40*μ*g ml^−1^ and (f) 200*μ*g ml^−1^ of meropenem. These results are plotted along with the data points from data set 1 with errorbars displaying the standard deviation of the means of three biological replicates. Data values are measured in optical density (OD); we formulate the solution for OD using the equation $OD(t)=H(t)+\frac{S(t)}{2}$.

Using parameter sets Θ_1_ and Θ_2_ we obtain the results shown in Fig 4; both parameter sets successfully fit the model to all of the experimental data. The total errors of these data fits over all concentrations are 3.47 × 10^−3^ and 3.56 × 10^−3^ respectively and if taken as an indicator of goodness of fit, along with the visual fits shown, we see that there is little difference in the ability to model this system using either parameter set.

**Fig 5:**
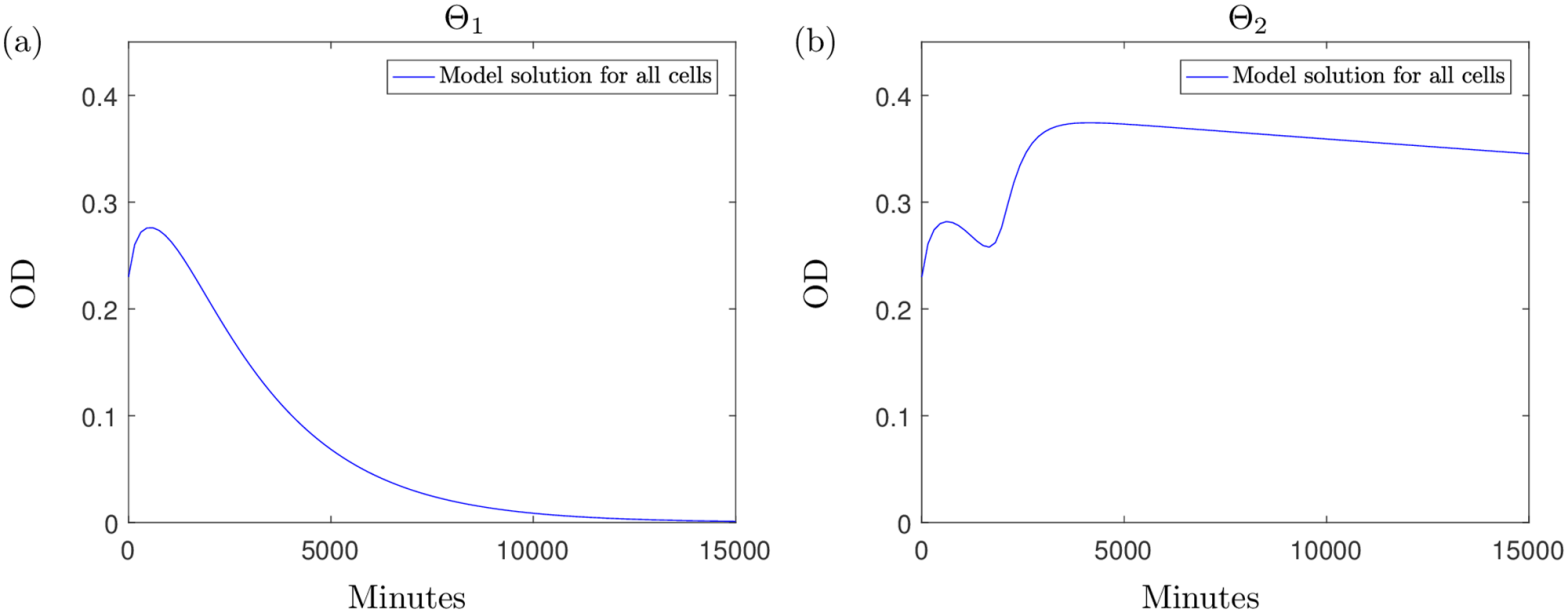
The long term solution to the system (2)–(6), using parameter sets (a) Θ_1_ and (b) Θ_2_ from Table 3, with *A*_0_ = 2. Parameter set Θ_2_ has a faster rate of antibiotic loss due to internalisation and this results in the antibiotic degrading from the system before it can sufficiently eliminate the bacteria. For higher concentrations of antibiotic we see the bacteria being eliminated by the antibiotic for both parameter sets (results omitted).

**Fig 6:**
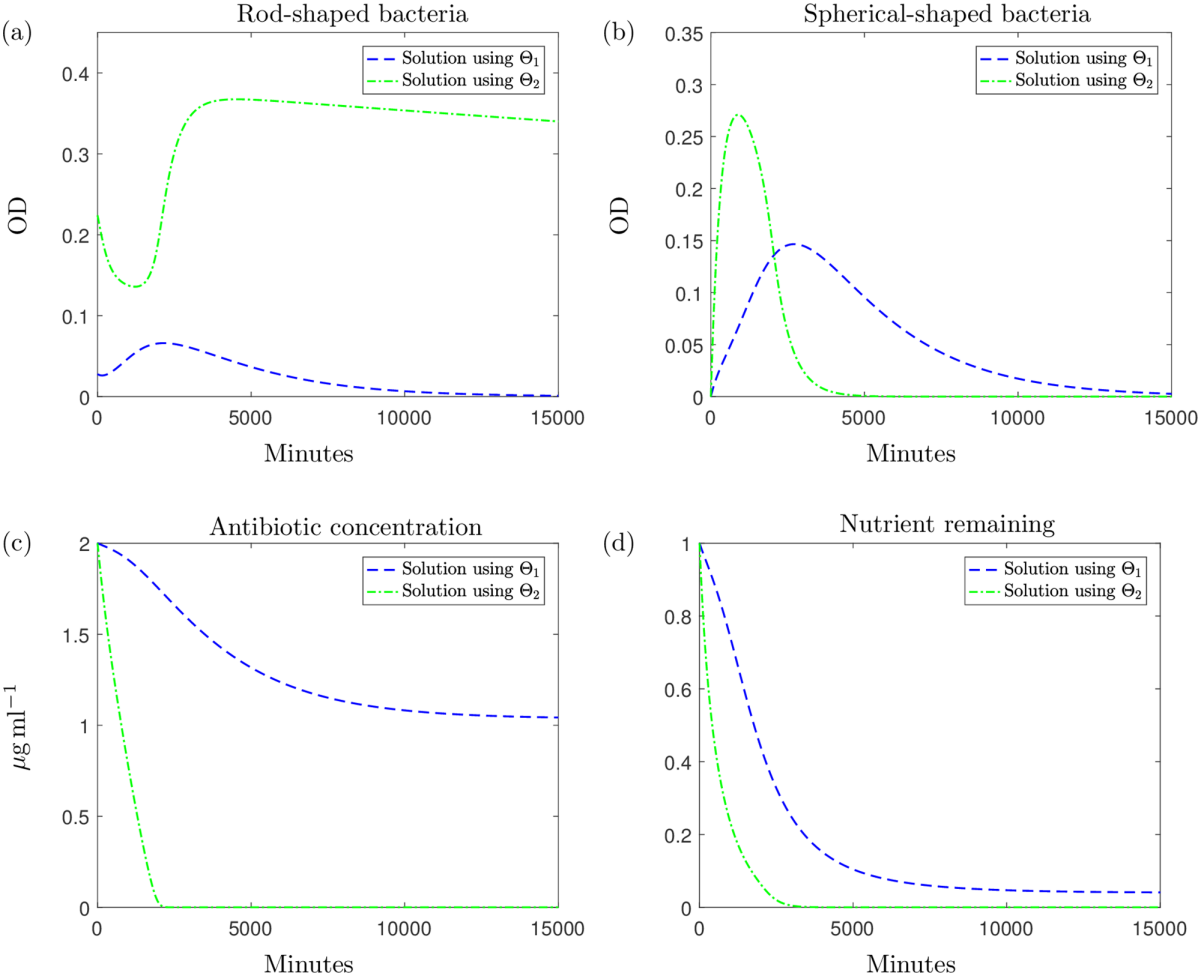
The individual variable solutions to system (2)–(6) with *A*_0_ = 2. The solution using Θ_1_ is represented with a blue dashed line and the green dashed-dotted line represents the solution using Θ_2_ for (a) rod-shaped cells, (b) spherical shaped cells, (c) antibiotic concentration and (d) nutrient. We note that these solutions are simulated over a longer time series than the experiments. The results for higher concentrations of antibiotic follow the trend of Θ_1_ for each variable when using either parameter set. The only exception to this is the predicted growth of *H* when using Θ_2_; here the population of *H* is eradicated to low OD values for all higher values of *A*_0_.

The qualitative differences between Θ_1_ and Θ_2_ emerge when we use the model to predict the population growth over a longer time only for the case when 2*μ*g ml^−1^ is added (for higher concentrations population eradication occurs for both parameter sets). Fig 5(a) displays the model solution for the predicted growth of the bacterial population over a time of approximately 10 days using parameter set Θ_1_: the model predicts the eventual elimination of the population due to antibiotic effects and an exhausted nutrient supply. In contrast, if we simulate the experiment for this time using Θ_2_, we get the results shown in Fig 5(b). The results indicate that instead of the population dying out, we would expect a large growth recovery period shortly after the time of the experiment, with the optical density levels surpassing those measured in the experiments. This is followed by a slow, natural death phase caused by nutrient depletion. To explain this, we break down the solution in Fig 6(a)–6(d). These single variable solutions show that when using Θ_2_, the antibiotic degrades at a higher rate than under parameter set Θ_1_. Due to the higher antibiotic depletion rate, we predict that the antibiotic will be unsuccessful in killing off the bacteria and growth resumes until around 5000 minutes when nutrient supply depletes and slow natural death dominates. The nutrient levels predicted using Θ_1_ indicate that the nutrient is not completely used up but due to the high level of remaining antibiotic, bacterial growth cannot exceed death and this results in the population being eradicated by the antibiotic. We also notice, when modelling the system using Θ_2_, that the population of spherical cells depletes entirely, shortly after the antibiotic is expended: the cells revert back to the native bacillary morphotype, as witnessed in the results in [8] and our microscopy results in Fig 1.

These qualitatively different characteristics result from varying values of only two parameters and indicate the sensitivity of the model solution to these parameters. Furthermore, the differences in parameter values indicate that it is the value of $\tilde{\rho }$ that determines whether we predict population eradication or recovery. Θ_1_ has a slower rate of natural antibiotic degradation, *α*, yet it is possible to use a higher value for this parameter and this still results in the population dying out sooner (results omitted). The difference in our values of $\tilde{\rho }$ is less significant numerically than that for *α* yet drastically changes the estimated growth characteristics of the bacteria.

### Parameter testing

#### Testing OD predictions

**Fig 7:**
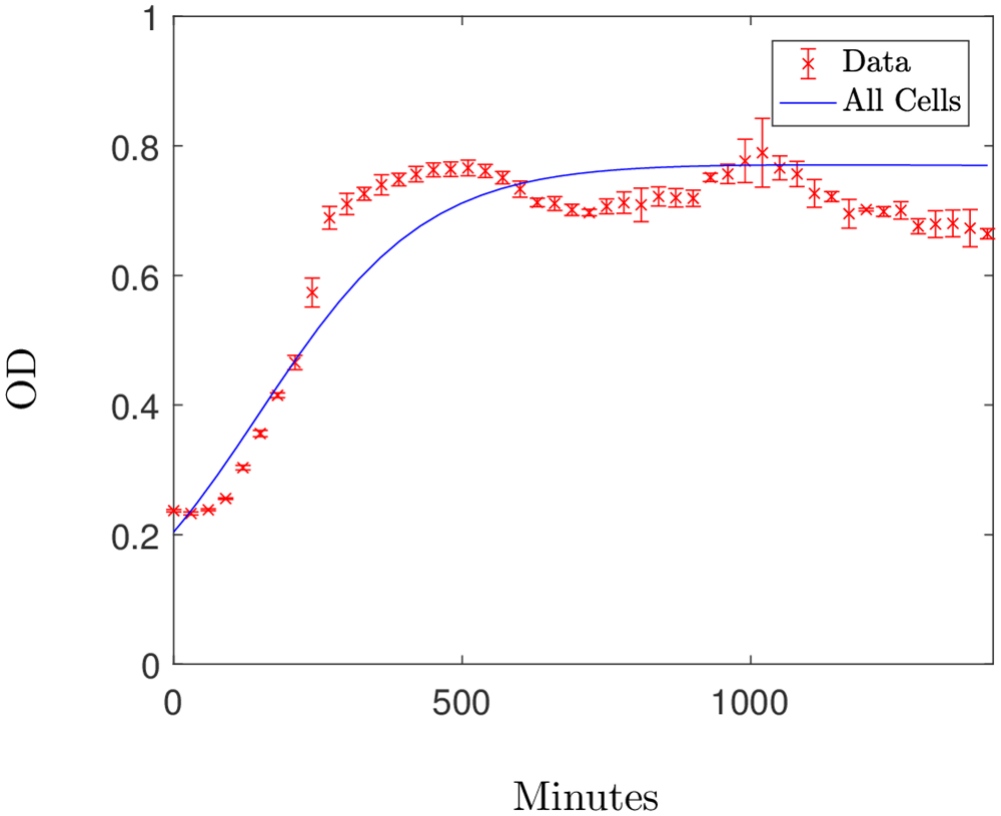
The solution to the system (7)–(9) using the parameters in (10), plotted with the additional test data for the growth curve of *P. aeruginosa* (Data set 2). Using *fminsearch* we get a relative error value of *R* = 0.002 with an estimated initial condition *H*_0_ = 0.2 when using growth rates (10).

To test our parametrisation results, we use data set 2 to examine whether the parameters obtained can successfully fit the data acquired from an additional biological replicate, with differing initial antibiotic concentrations. Initially we tested the parameters obtained using the growth curve in the absence of meropenem; by fixing these parameters yet allowing the initial condition to be fitted to the new data, we are able to successfully describe the growth curve of the new data. The results, shown in Fig 7, show that although we are not able to capture all the detailed dynamics of growth, the growth rates found for the initial data set are able to predict the overall behaviour of the new data set when no antibiotic is added.

**Fig 8:**
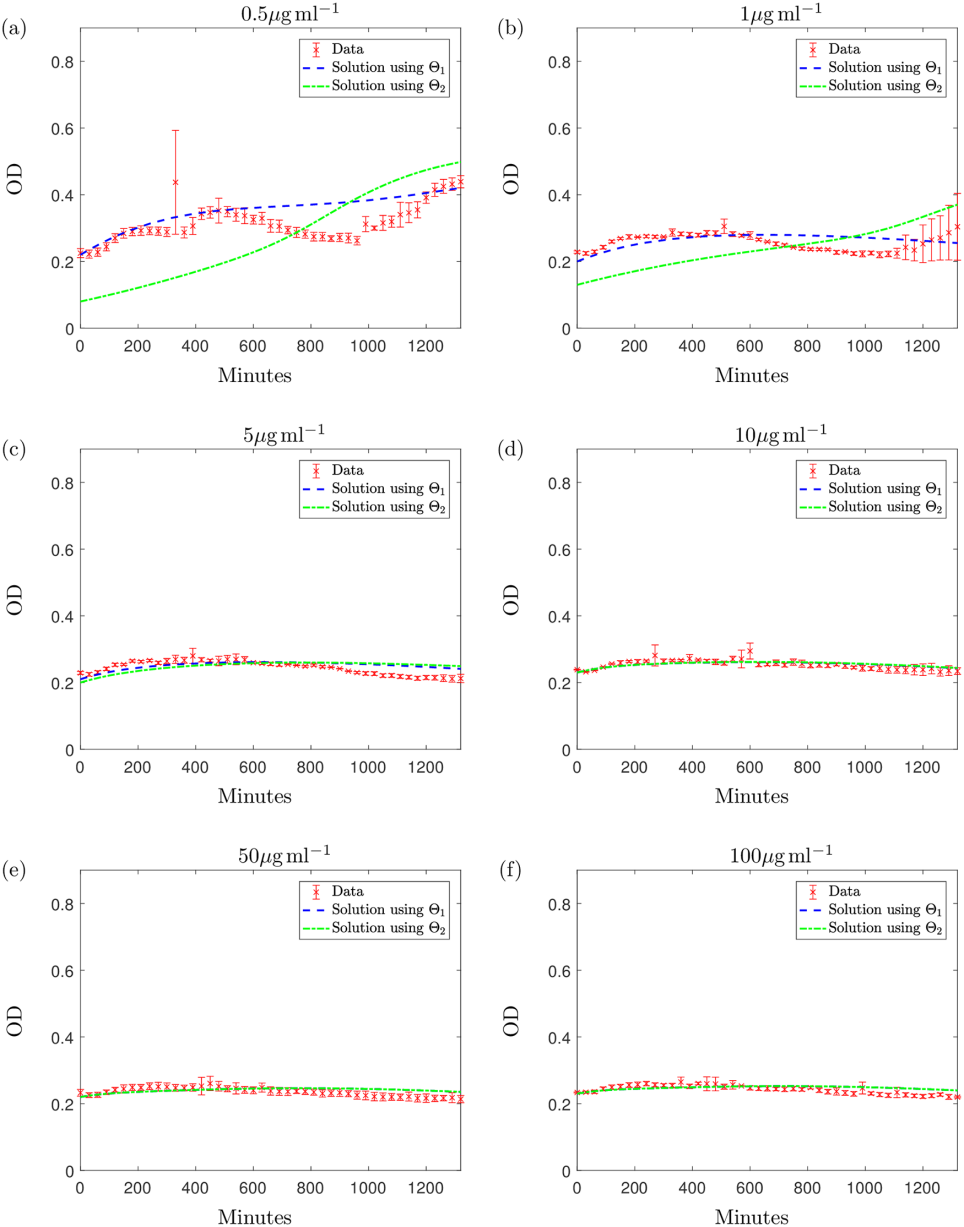
The solution to the system (2)–(6) with: The solution using Θ_1_ is represented with a blue dashed line and the green dashed-dotted line represents the solution using Θ_2_. These results are plotted along with the data points from data set 2 (along with standard deviation errorbars) with initial antibiotic concentrations of (a) 0.5*μ*g ml^−1^, (b) 1*μ*g ml^−1^, (c) 5*μ*g ml^−1^, (d) 10*μ*g ml^−1^, (e) 50*μ*g ml^−1^ and (f) 100*μ*g ml^−1^ meropenem. The initial conditions were estimated using the initial data points as initial estimates. Data values are measured in optical density (OD); we formulate the solution for OD using the equation $OD(t)=H(t)+\frac{S(t)}{2}$.

After testing Θ_1_ and Θ_2_ against the new data set that include the addition of antibiotic we obtain the results displayed in Fig 8. Although both parameter sets are suitable in describing the general trend of the curves for the higher concentrations of antibiotic (≥2*μ*g ml^−1^), Θ_1_ describes the dynamics of the new kill curve data at the lower concentration more successfully. Θ_1_ gives a relative error value of *R* = 0.014 and the model captures the general trend of each curve at the earlier and later timepoints but does miss some qualitative behaviour at intermediate timepoints (the dip in OD prior to regrowth observed in Fig 8(a)–8(c) does not emerge). Nevertheless the model fits the test data (including new concentrations) sufficiently well to proceed to the subsequent analysis of the parametrised model. We note that the initial conditions have been fitted to the data; fixing the initial conditions results in a similar prediction for Θ_1_ and using Θ_2_ the model overestimates growth.

Additionally, we notice that the experimental results using 0.5*μ*g ml^−1^ and 1*μ*g ml^−1^ display periods of population regrowth at later timepoints, a result that is also seen in the microscopy results at 22 hours (see Fig 1). The model predicts this behaviour (over a longer timeframe) when using Θ_2_ for an initial concentration of 2*μ*g ml^−1^. Using antibiotic concentrations from data set 2, Θ_1_ can also successfully describe the post-antibiotic regrowth phase for lower concentrations, as well as predicting the eradication of the population using higher concentrations.

Testing the parameter sets against this new data suggests Θ_1_ may be a more viable parameter set but importantly we reiterate the dependence of these fits on the values of the unidentifiable parameters related to antibiotic decay. Furthermore, we shall see below that when comparing against a different experiment, Θ_2_ may match the data better.

Using data set 2 has enabled us to test the OD predictions of the model, however, the OD measurements provide no insight into the proportions of rod- and spherical-shaped cells within the total population. In order to validate our model predictions further we use microscopy results to estimate the contribution of each subpopulation to the overall OD value.

#### Calibration against microscopy images

The images in Fig 1 give glimpses of the bacterial population at three time points of the experiment and we can clearly see that a shape transition is occurring during the experiment. The proportion of different phenotypes visible in further images under 3 conditions and at 3 timepoints were obtained using the cell counter plug-in for Fiji [38, 39]. Visible in many of the images were cells that were of an intermediate form or filamentous and following the assumptions of the model, bacteria of all phenotypes that were not completely spherical were contained in the rod-shaped population. For each concentration and time point we obtained up to 7 images; Table 5 displays the mean proportions of each of the subpopulations over the various images used.

**Table 5:** The average proportions of each bacterial subpopulation, obtained using the microscopy images.

Antibiotic concentration	Subpopulation	Time *(Number of images)*		
		1 hour	6 hours	22 hours
0 *μ*g ml^−1^	*H*	1 *(5)*	1 *(6)*	1 *(7)*
	*S*	0 *(5)*	0 *(5)*	0 *(5)*
0.5 *μ*g ml^−1^	*H*	0.981 *(6)*	0.781 *(6)*	0.989 *(6)*
	*S*	0.019 *(6)*	0.219 *(6)*	0.011 *(6)*
2 *μ*g ml^−1^	*H*	0.95 *(5)*	0.411 *(5)*	0.953 *(6)*
	*S*	0.05 *(5)*	0.589 *(5)*	0.047 *(6)*

The values in this table were obtained by counting the number of cells in each subpopulation in each slide. The proportions were then calculated by dividing the number of cells in a each subpopulation by the total number of cells in the image. The mean proportion was then taken.

**Fig 9:**
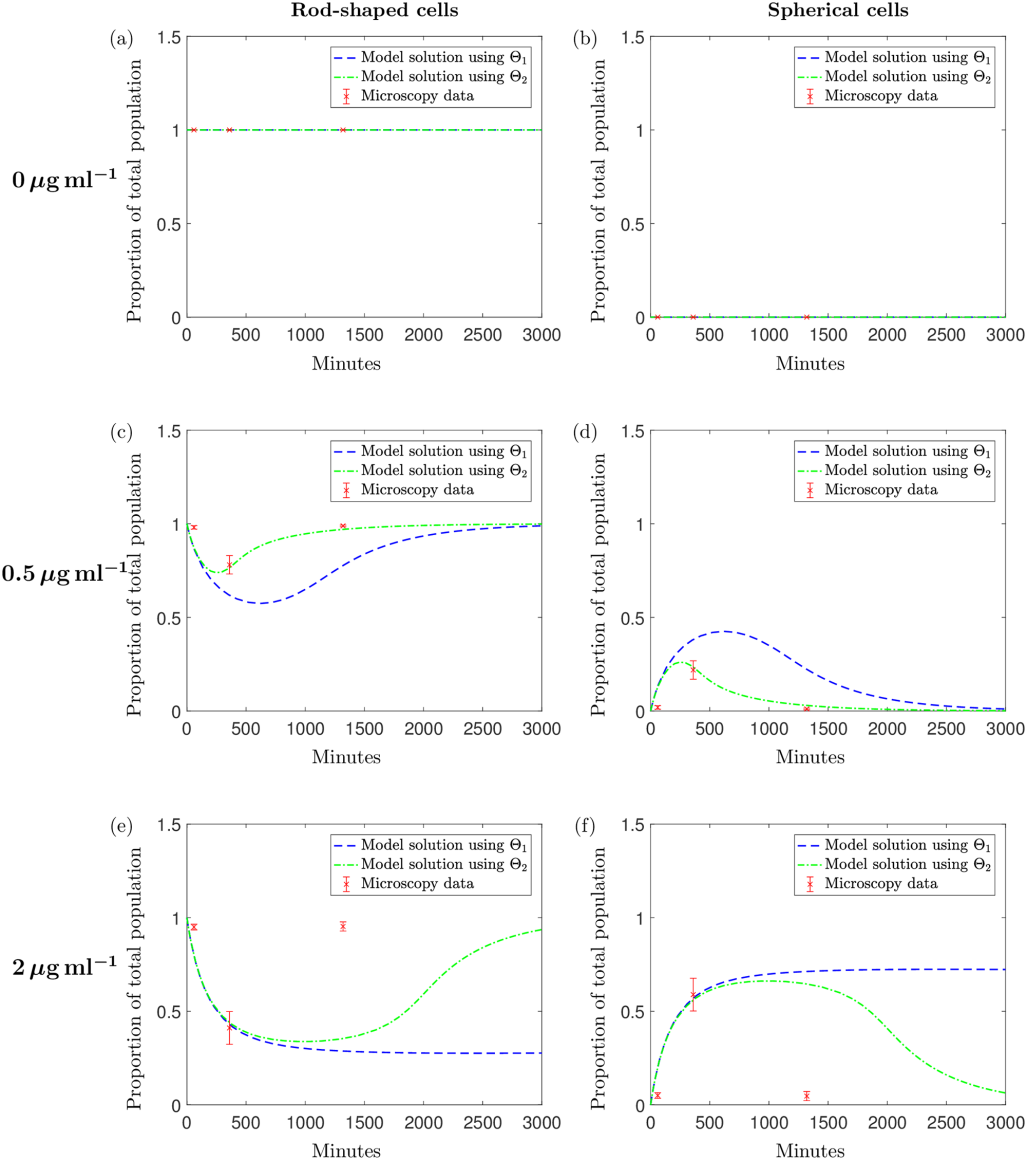
Comparing the predicted variable solutions to the data obtained from the microscopy images. The variable solutions with respect to total population for *H* with (a) *A*_0_ = 0, (c) *A*_0_ = 0.5, (e) *A*_0_ = 2, and *S* with (b) *A*_0_ = 0, (d) *A*_0_ = 0.5 and (f) *A*_0_ = 2. Solutions were obtained by solving the system (2)–(6) using Θ_1_ (blue dashed) and Θ_2_ (green dashed-dotted). The red markers show the proportion of each subpopulation with respect to the total number of cells counted in the microsocpy images. The errorbars indicate the standard deviation over the multiple images used.

To compare the cell count results to the model solutions, the model solutions must be scaled from OD to cell number. We use the scaling given in [40] where an OD measurement of 1 is given as equivalent to 2.04 × 10^8^ cells. Comparing the model predictions to the cell count data gives the results displayed in Fig 9. For the case when no antibiotic is added we see that the microscopy results match the model predictions exactly; since no antibiotic is in the system we would not expect the bacteria to transition and therefore we only see and predict rod-shaped cells. As we add antibiotic, we find that the microscopy counts match the model predictions well for the majority of timepoints, especially when using Θ_2_. Qualitatively, for both parameter sets (and quantitatively for Θ_2_), we get a good comparison for the lower concentration of 0.5*μ* g ml^−1^; less than half of the bacteria transition to the spherical form over the first few hours of the experiment before the population becomes almost entirely comprised of rod-shaped cells towards the end of the experiment. As we increase the antibiotic concentration we get a very good comparison over the first two timepoints but at the final timepoint we notice some disparity between the cell counts and the model solutions. Qualitatively, the solution using Θ_2_ is equivalent to the microscopy result. Θ_1_ predicts different qualitative behaviour with a long-term persistent population of spherical cells. At higher concentrations of meropenem, both parameter sets predict this and this is reflected in the killing curve data where significant growth does not occur at these higher concentrations. Qualitatively the experimental data shows the spherical cells transitioning back to rod-shaped more quickly than that captured by Θ_2_. However, the fragility of the spherical cells will influence the experimental result: in [8] it was found that spherical cells would easily burst when placed directly between the slide and coverslip. It is likely that this also would have occurred during our experiments, thus leading to fewer spherical cells being visible in the images. It is therefore possible that the population appears to transition back to fully rod-shaped quicker than it actually would since many of the spherical cells in the sample could burst before the image was taken.

Given the good qualitative and quantitative agreement between the two types of data sets and both Θ_1_ and Θ_2_, we proceed our analysis using both parameter sets. Recall that the difference between the two data sets results from two unidentifiable parameters; continuing with both parameter sets we can ensure a comprehensive range of plausible qualitative behaviour is explored.

### Manipulating the morphological transition

Our primary interest in formulating this model is to investigate the proposed morphological change observed in our experimental data of *P. aeruginosa*. This shape transition has been associated with the ability of the bacteria to withstand high levels of antibiotic and using suitable parameters we can reproduce the bacterial regrowth witnessed in our experimental data. We can now manipulate the system by changing parameters associated with the transition mechanism to investigate the impact the shape transition has on bacterial susceptibility and produce predictions that could facilitate treatment design.

**Fig 10:**
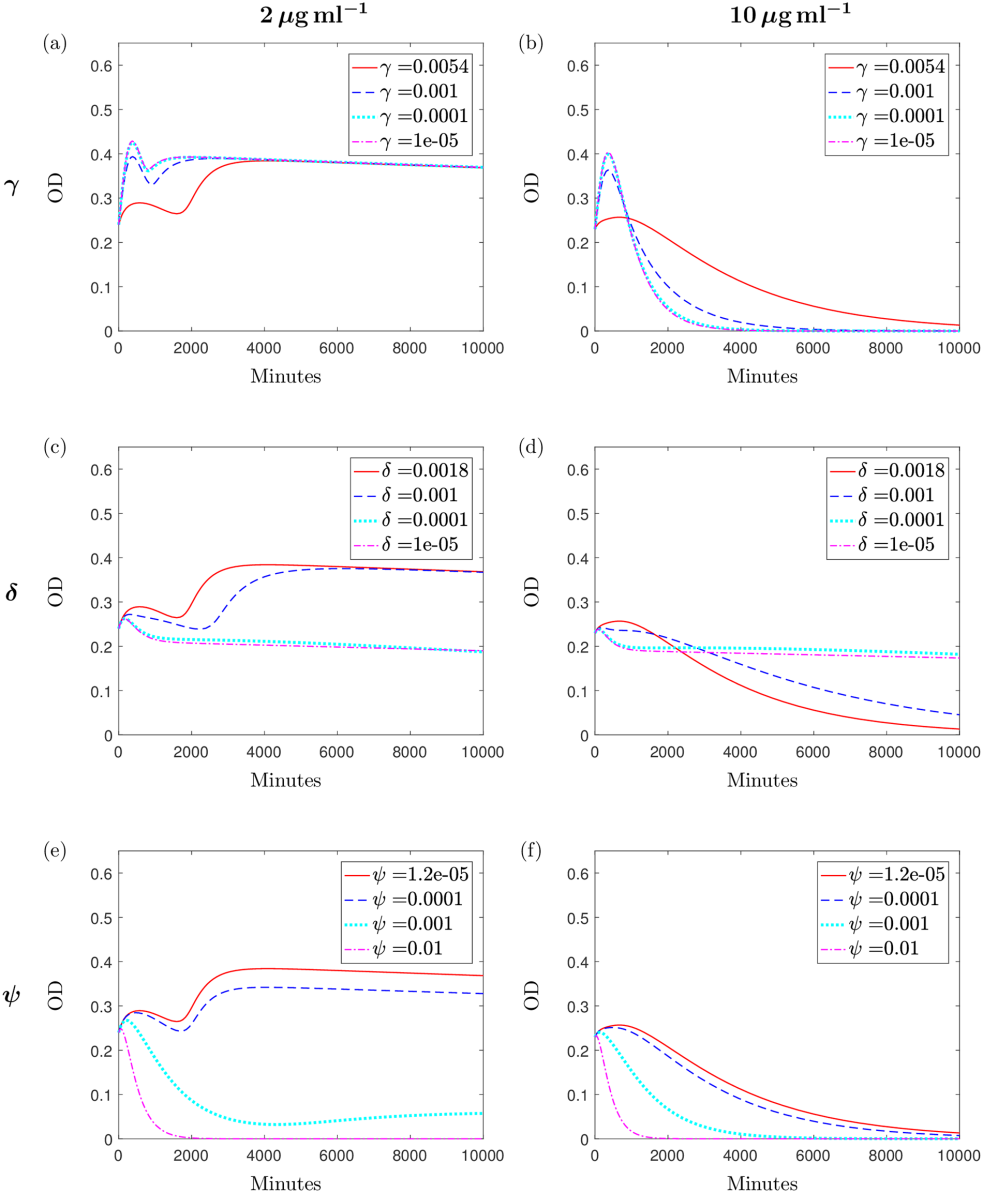
Simulations displaying predictions of bacterial growth with variations to key parameter values. The solution to the system (2)–(6) using Θ_2_ and varying the parameters (a) *γ* for the case where *A*_0_ = 2, (b) *γ* for the case where *A*_0_ = 10, (c) *δ* for the case where *A*_0_ = 2, (d) *δ* for the case where *A*_0_ = 10, (e) *ψ*, when *A*_0_ = 2 and (f) *ψ* when *A*_0_ = 10. The default values, found via paramterisation, are depicted by the red solid lines. Values for all varied parameters have units min^−1^.

Fig 10(a)–10(f) display numerical simulations used for the analysis in this section. We note here that the results using Θ_1_ are qualitatively the same regardless of the initial antibiotic concentration and these are also similar to the results when using Θ_2_ with initial antibiotic doses of >2*μ*g ml^−1^. The results for Θ_2_ differ for higher (>2*μ*g ml^−1^) and lower (2*μ*g ml^−1^) antibiotic concentrations and therefore we only include simulations using Θ_2_ for brevity (i.e. the Θ_1_ results are equivalent to the Θ_2_ results at 10*μ*g ml^−1^. We have chosen to display simulations using the initial antibiotic dose of *A*_0_ = 2*μ*g ml^−1^ as the results in this case differ from the other concentrations and *A*_0_ = 10*μ*g ml^−1^ as this case represents a clinically relevant antibiotic concentration.

#### Inhibiting the change from rod-shaped to spherical-shaped cells

To propose that the morphological transition is a defensive mechanism that occurs due to antibiotic exposure would imply that inhibition of this mechanism would be detrimental to the bacteria. By decreasing the transition rate *γ* we can examine whether inhibition may lead to enhanced antibiotic action and scrutinise the impact of the transition mechanism.

Fig 9(a) and 9(b) illustrate numerical simulations of the experiment varying *γ* using Θ_2_ and initial antibiotic doses of 2*μ*g ml^−1^ and 10*μ*g ml^−1^ respectively. At 10*μ*g ml^−1^, these simulations imply that inhibiting the transition to a spherical morphology would initially lead to higher OD levels but in all cases we ultimately achieve increased antibiotic action with the bacterial population being eradicated faster than when we use the default value of *γ* from the respective parameter set. The higher initial OD levels are a consequence of having more rod-shaped cells in the system as they have a higher contribution to the OD than the spherical cells.

A different result is obtained in Fig 9(a). In this case we see that though a decrease in *γ* will result in an increase in OD levels initially (as above), the population will grow to the same value regardless of the decrease in transition rate. This population value is reached once the antibiotic dose of 2*μ*g ml^−1^ has been exhausted and the system is reduced to Eqs (7) and (8). Bacterial growth once again becomes dependent on the proportion of nutrient remaining in the system and as this depletes, bacterial growth is unable to exceed bacterial death, resulting in a post-antibiotic, post-nutrient death phase. Thus the transition to spherical cells may be considered a long-term defence mechanism only at higher concentrations of antibiotic.

#### Inhibiting the reverse transition

Our microscopy results support the conclusion made by Monahan *et al*. that *P. aeruginosa* bacteria are able to transition back to rod-shaped cells after transitioning to a spherical form [8]. This reverse transition grants the bacteria the ability to resume growth and in Fig 6(a) and 6(b) we saw results that indicate the impact of this mechanism on restoring the bacterial population size. Decreasing *δ*, the transition rate from spherical to rod-shaped bacteria, from its estimated value illustrates how inhibiting this mechanism would impact the survival of the bacteria.

Fig 10(c) and 10(d) show the results of these simulations. Our variable solutions (omitted) indicate that for all initial antibiotic concentrations and using both parameter sets, decreasing the rate of the reverse transition predicts a higher population of spherical cells and fewer rod-shaped cells, as might be anticipated. If we inhibit the transition sufficiently (*δ* < 1 × 10^−4^ min^−1^), the model predicts similar overall population levels (OD_600_ values of around 0.2), that are made up entirely of spherical cells, regardless of the parameter set used or the initial antibiotic concentration (compare the blue and pink lines in Fig 10(c) and 10(d)). Since we have assumed that the spherical cells cannot proliferate but do evade antibiotic effects, the larger spherical cell population results in prolonged antibiotic and nutrient availability. Consequently, this predicts a system where though spherical cells can transition back to a rod-shape at a much slower rate in doing so, they become exposed to the effects of the unused antibiotic. Alternatively, they die naturally due to their inherent fragility and this produces an extended death phase that ultimately leads to population eradication, but over a much extended period of time than simulated. Conversely, if the level of inhibition is not sufficient then we predict lower population levels initially than with default parameters, although behaviour is the same over the prolonged period of time.

In cases where the antibiotic was previously unable to eradicate the population (for example when we simulate the case of 2*μ*g ml^−1^ using Θ_2_), inhibiting the transition at which spherical cells can revert back to the bacillary form is advantageous to us as it extends the efficacy of the antibiotic. This is reflected in Fig 10(c) where we see that the population level predicted through sufficient inhibition is less than that predicted when using the default value. However, Fig 10(d) displays the results when we use parameter set Θ_2_ and let *A*_0_ = 10*μ*g ml^−1^; this represents a system that would have previously predicted population eradication and by inhibiting the transition, we see that the total population level achieved through sufficient inhibition is larger than we predicted when using the default parameter value. Restricting the reverse transition interferes with the antibiotic action and prevents the antibiotic from killing the bacteria. This result is reiterated when using Θ_1_ for all initial concentrations and Θ_2_ for concentrations above 2*μ*g ml^−1^ (results omitted for brevity).

#### Investigating the use of antimicrobial peptides

In addition to investigating and establishing the existence of the transition mechanism, Monahan *et al*. experimented with the use of antimicrobial peptides (AMPs) as an additional antimicrobial agent. These AMPs are relatively inactive against rod-shaped *P. aeruginosa* as they induce cell death by creating pores in the cytoplasmic membrane, which remains unexposed in normal bacillary cells. However, they found that spherical cells displayed large areas of exposed cytoplasmic membrane which gave reason to believe that they would be more susceptible to the bactericidal effects of AMPs. This hypothesis was supported by experimental data that saw a combination therapy of meropenem and AMPs leading to lower bacterial load [8].

We can reproduce this theoretically by increasing the death rate of spherical cells, *ψ*. Increasing the death rate *ψ* is equivalent to administering AMPs; these results are shown in Fig 10(e) and 10(f). An expected increase in spherical cell death and consequently a decrease in rod-shaped cells results in faster population suppression for all concentrations and both parameter sets. The depleted spherical cell population means that fewer cells transition back to their native bacillary form and lower OD levels are achieved overall. We note that for the case of 2*μ*g ml^−1^ using Θ_2_ *ψ* must be increased from its default value more than is required for the other cases for the AMPs to successfully assist the meropenem in killing off the bacteria population, in comparison to cases with higher initial antibiotic concentration or using Θ_1_. However, under all parameter conditions, any increase in spherical cell death results in lower OD predictions and this reinforces the results of Monahan *et al*. and advocates the use of meropenem in combination with AMPs as a potential therapy for treating *P. aeruginosa*.

## Discussion

Following the conclusions made by Monahan *et al*. and supported by our own data, we have formulated a model that describes the growth dynamics of *P. aeruginosa* in the presence of meropenem, with the inclusion of a sub-population of cells with an incomplete cell wall that results in an altered shape and size. We have made assumptions regarding the inducement of this morphological shift and by successfully fitting it to experimental data we obtained two parameter sets that successfully fit the data yet result in qualitatively different predictions of the long-term bacterial dynamics and different predictions when we investigate the values of some of the model parameters.

We can attempt to address the ambiguity surrounding the cause of structural changes to the bacterial population using this model. By assuming that the change in bacterial morphology occurs due to the presence of antibiotic exposure, whether the transition is an intrinsic internal response of the bacteria or a result of antibiotic action, the model formulated allows for both of these possibilities.

Research suggests that the formation of spheroplasts, with a depleted peptidoglycan layer and unstable osmotic properties, may be the final structural change witnessed before lysis, implying that the transition may be due to antibiotic effects and not an intrinsic mechanism. Although this hypothesis may contradict the findings in [8], the model is still suitably formulated under this assumption. The death rate of the bacillary cells due to antibiotic can be seen to represent those cells that are eliminated by the antibiotic before any significant changes in morphotype occur. Those that do display changes in their ultrastructure undergo transition into the spherical population and are then subject to a higher death rate; however this mechanism is not explicitly dependent on antibiotic. Another way of looking at this would be to suggest that if the shift in population structure is solely the result of peptidoglycan inhibition, induced by the antibiotic, then once a bacterium has converted into a spherical form the bactericidal damage has already been induced and lysis is no longer depend on antibiotic presence. Similarly, allowing for a reverse transition would suggest that the damage made by the antibiotic is reversible.

Much of our results, however, support the hypothesis proposed by Monahan *et al*. [8] that this transition is in fact a purposeful evasion mechanism, induced by antibiotic exposure, yet ultimately implemented by a responsive cellular mechanism. Under the assumption that spherical cells are immune to antibiotic effects, and by using suitable parameters, we have shown that the transition may lead to the recovery of population levels in the absence of meropenem, which we have seen in both our microscopy results and growth curves using low antibiotic concentrations. Although these results are restricted by limited data, the repercussions of this theory could give insight into the persistent characteristics of *P. aeruginosa* and explain its ability to sometimes withstand high levels of carpabenems. This could be seen as a characteristic of resistance that is displayed intrinsically without any reliance on the inheritance of resistance genes or acquisition of specific mutations that warrant resistance to the antibiotic. Owing to this, we must consider the possible impact of the shape transition in the wider context of antibiotic resistance; if intrinsic in nature then the use of this mechanism could work hand-in-hand with other resistance mechanisms. Additionally, possessing a mechanism that causes this kind of morphological shift can be linked with persistence and the threat of recurrent infection that comes along with phenotypic persistence.

By formulating a mechanistic model we have obtained parameter values that are not only potentially measurable but are also more meaningful and directly relate to the amount of nutrient in the experiment. The model can successfully describe the overall trends witnessed in the growth curves and although the model does not capture the growth dynamics over the first few hours of the experiment perfectly, the impact of this on the applications of this model is arguable as our interests lie with the long term predictions of population growth, which are accurately predicted. The error over this region increases for the higher antibiotic doses yet we note these are much higher than the recommended clinical dose of 10−20*μ*g ml^−1^, therefore any discrepancies obtained for these concentrations should have no clinical relevance. We also take note of the large error bars for the initial condition: the high level of variance between the measurements taken at this time point means it could be difficult to achieve a fit of high confidence in this region regardless. Furthermore, having parametrised the model from total bacterial load (OD measurements) the model is then able to correctly predict the underlying dynamics of the individual rod- and spherical-shaped populations (calculated from the microscopy data).

Manipulation of the parameters obtained allows us to investigate the impact of inhibiting the transition mechanism. Our results suggest that inhibiting the spherical transition would be detrimental to bacterial populations in most cases with inhibition leading to faster depletion and lower OD levels over a long time scale. An exception to this is when we use parameter set Θ_2_ and initial antibiotic concentration of 2*μ*g ml^−1^; in this case, inhibition is only detrimental to growth in the short term, whereas long term predictions are the same regardless of the level of inhibition. In most cases (including all antibiotic concentrations for Θ_1_) the results imply that inhibition of the spherical transition would be desirable in treating a *P. aeruginosa* infection and thus support the hypothesis that this may be an intrinsic defence strategy of the bacteria.

When the transition to spheres was inhibited, the simulations also displayed a characteristic of higher OD levels over the first few hours than with no inhibition; if these results were to be directly translated to an *in vivo* infection, this could imply a higher level of bacterial virulence. However, in order to formulate biological conclusions based on these results we must consider the virulent properties of the different cell types we include in the model. A higher OD value does not automatically signify a higher level of virulence since we cannot assume that the virulence of spherical cells is half that of the rod-shaped cells, an assumption we make for the OD. Virulence in *P. aeruginosa* is to a large extent based on the Type III secretion system and secreted toxins; both of which depend on outer membrane components for successful secretion. It is probable that the spherical cells are less virulent than the rod-shaped cells due to their compromised outer membrane. If this assumption is correct then although inhibition of the shape transition predicts higher antibiotic action over the total time, a higher initial bacterial load would not be a desirable effect of a treatment when these are predominantly rod-shaped cells. Increased virulence could be threatening to a patient’s health and this would be especially dangerous when treating immunocompromised patients.

It is also worth noting here that if the morphological changes witnessed are a result of antibiotic action then inhibiting this mechanism could be counter-productive to the application of meropenem. Envisaging a drug that could inhibit the bacterium from shedding the cell membrane, thus keeping it intact, this could work directly against the mechanism of action of the antibiotic.

An alternative strategy would be to target the reverse reaction and our results indicate that this approach could be beneficial, depending on the virulent properties of the spherical cells. By inhibiting this reaction we predict a higher proportion of spherical cells and this enables successful suppression of a lower proportion of remaining rod-shaped cells without exhausting the antibiotic supply. Following this phase of rod-shaped cell death, the model predicts that for sufficient inhibition, the bacteria attains similar spherical-only population levels regardless of the parameter set used or initial antibiotic concentration. Though in many cases the estimated population level is higher than we would predict using the default parameter values, this would not be an undesirable outcome under the assumption that spherical cells are not as virulent as rod-shaped cells due to the compromised cell membrane.

Owing to the high proportion of spherical cells we predict when inhibiting the spherical to rod transition, we could consider the combination therapy of meropenem and AMPs with a drug that would inhibit the reverse transition. Simulations suggest that if we sufficiently inhibit the reverse transition rate then any increase in spherical cell death would result in faster killing of the bacterial population and extended antibiotic presence regardless of the parameter set used or initial antibiotic concentration (results omitted for brevity). Here we define sufficient inhibition of the reverse transition to be when we choose a value for *δ* that predicts population levels comprised of mostly spherical cells.

If we do not attempt to inhibit the morphological transition and instead pursue the application of a drug that increases the death rate of spherical cells alone, we predict lower OD levels. This supports the results detailed in [8] that promote the use of AMPs as a supplementary agent in combination therapy with the meropenem and empiric studies have also found that *β*-lactam and AMPs act synergistically to inhibit growth [41]. This could be a very suitable strategy for treating a *P. aeruginosa* infection, however further investigation into the resultant impact this may have on the emergence of resistance would be needed. The use of AMPs would impose a direct fitness cost on the spherical cells and if the transition occurs as a result of an intrinsic mechanism then this could impact selection for resistant phenotypes. However, due to the already-depleted cell wall and lowered natural survival rate of the spherical cells, it is uncertain whether imposing a further fitness cost on the bacteria would impact the possible emergence of resistance. The same evaluation would need to be made if a strategy targeting inhibition of either the forward or reverse transition was pursued; any target that results in lowered fitness could influence the selection for resistant phenotypes. Additionally, if we were to inhibit the rod to spherical shape transition, resistance would imply a bacterium not transitioning to a spherical form and this would imply that it would maintain a rod-shape morphology and remain susceptible to the antibiotic. Using an extended model it is possible to simulate the system with multiple strains (i.e. meropenem-resistant and -susceptible strains, similar to [14]) of the bacteria and we are currently investigating whether or not a resistant strain would flourish in this environment.

We have formulated a model to predict the growth dynamics of *P. aeruginosa* witnessed *in vitro*. In order to make conclusions in a clinical setting we would have to translate the model to an *in vivo* model and extend it to include antibiotic dosing instead of the single antibiotic application used in our experiments. We would also need to consider how nutrient availability would differ *in vivo*. Nutrient availability *in vivo* is likely to be lower than in the nutrient rich growth media used in experiments, but it may be replenished over time. Numerical simulations (omitted) indicate that if nutrient-abundance is maintained for longer, the bacteria may withstand higher concentrations of antibiotic.

In conclusion, by formulating a model based on biological mechanisms, including a morphological transition, into a mathematical model for population growth, we have successfully obtained parameters values that describe the rates of the mechanisms involved. Crucially, extending this model will allow for a better prediction of how these potential therapies may impact the emergence and development of drug resistance and parameter analyses could hint at strategies to combat the threat of resistance. Our analysis suggests that inhibition of the morphological transition could be a suitable target for a treatment strategy.
